# The Role of Dietary Supplements in the Treatment of Endometriosis: A Critical Review

**DOI:** 10.3390/nu18081274

**Published:** 2026-04-17

**Authors:** Mariusz Wójtowicz, Paweł Małek, Magdalena Olszanecka-Glinianowicz

**Affiliations:** 1 Clinical Department of Gynecology and Obstetrics, Faculty of Medical Sciences in Zabrze, Medical University of Silesia, 40-055 Katowice, Poland; mariuszwojtowicz007@onet.eu; 2 Health Promotion and Obesity Management Unit, Department of Pathophysiology, Faculty of Medical Sciences in Katowice, Medical University of Silesia, 40-055 Katowice, Poland; pmalek@sum.edu.pl

**Keywords:** endometriosis, dietary supplements, treatment

## Abstract

**Background:** There is a growing number of studies suggesting the effectiveness of dietary supplements in preventing and treating endometriosis. It has been suggested that deficiencies in vitamins D and E as well as zinc are associated with the increased risk of endometriosis development. Beneficial effects of magnesium, curcumin, resveratrol and epigallocatechin-3-gallate were found in experimental animal studies. A reduction in pain related to endometriosis was shown in women using omega-3 and alpha-lipoic acid. Meanwhile, decreasing endometriotic lesion size after the supplementation of omega-3, *N*-acetylcysteine, vitamin C and epigallocatechin-3-gallate was observed in animal and human studies. Thus, the aim of this critical review was to summarize the available data describing the effects of dietary supplements used in the treatment of endometriosis. **Material and Methods:** The PubMed, Embase, Cochrane, and Web of Science databases were searched for related studies until 15 December 2025. Finally, 34 studies were included in the synthesis. **Results:** Of these 34 studies, only 23 were randomized, placebo-controlled trials. There have been no RCTs evaluating the effectiveness of vitamin E, zinc, alpha-LA, EGCG and DIM in the treatment of endometriosis. Single studies evaluating the effectiveness of vitamin C, magnesium, resveratrol, NAC and PEA with PLD have not confirmed it. Meanwhile single studies evaluating the effectiveness of selenium, propolis and quercetin have confirmed it. Of the four studies assessing the effectiveness of vitamin D, two confirmed it and two did not; of the two studies assessing probiotics, one confirmed its effectiveness and one did not; of the two studies assessing curcumin, one confirmed its effectiveness and one did not; and of the three studies assessing omega-3, two confirmed its effectiveness and one did not. All four RCTs assessing the combination of vitamins C and E confirmed their effectiveness. **Conclusions:** Despite encouraging observations from experimental studies, the results of RCTs are less encouraging and do not allow for the formulation of recommendations concerning the use of supplements in the treatment of endometriosis symptoms according to EBM.

## 1. Introduction

Endometriosis is a frequent cause of chronic pelvic pain in women and may be accompanied by infertility [1]. The symptoms of endometriosis depend on its localization: abdominal or extra-abdominal. The red flags of pelvic endometriosis include chronic pelvic pain with or without cyclic flares as well as gastrointestinal and urinary symptoms [2], while the symptoms related to extra-abdominal endometriosis are pain under the shoulder blade, catamenial pneumothorax, cyclical cough/hemoptysis/chest pain, and cyclical scar swelling/pain [3]. Although endometriosis occurs in 2–10% of women in reproductive age its pathogenesis is still not fully understood. The known mechanisms include uterine tissue damage or scarring; the uterine microenvironment; stem cells; remnant cells from menstrual blood; hormones; and genes products regulating inflammation, apoptosis, invasion, angiogenesis, autophagy, and oxidative stress [4,5,6]. It is believed that the key mechanism of the initiation and adhesion of endometriosis lesions as well as infertility and pain related to endometriosis is inflammation involving macrophages, natural killer cells, T cells, and dendritic cells regulated by cytokines, prostaglandins, and chemokines, and excessive estrogen levels [4,7,8,9].

There are no clear guidelines for the treatment of endometriosis. The recommendations of various scientific societies differ, albeit slightly. Most widely accepted guidelines include those of six nationals [College National des Gynecologues et Obstetriciens Francais, National German Guideline (S2k), Society of Obstetricians and Gynaecologists of Canada, American College of Obstetricians and Gynecologists (ACOG), American Society for Reproductive Medicine (ASRM) and National Institute for Health and Care (NICE)] and two internationals (World Endometriosis Society and European Society of Human Reproduction and Embryology). Most of these guidelines in the treatment of endometriosis-associated pain recommended progestins (dienogest or medroxyprogestetrone acetate) and combined oral contraceptive pills as first line therapy with a great evidence grade. As second line should be considered GNRH-agonists and levonorgestrel intrauterine system. The surgical excision of the endometrial implants and the endometriomas also plays an important role in the treatment of pain related to endometriosis. While in the treatment of infertility associated with endometriosis the recommended first line is excision of endometriomas and endometriosis, the second line therapy is ablation of the ovarian endometriosis. Pharmacotherapy is not recommended, except GNRH-agonist used prior to in vitro fertilization or surgery. None of the above societies recommend the use of dietary supplements in the treatment of pain and infertility associated with endometriosis [10]. However, there is a growing number of studies suggesting the effectiveness of dietary supplements in preventing and treating endometriosis. It has been suggested that deficiencies in vitamins D and E as well as zinc are associated with the increased risk of endometriosis development. Beneficial effects of magnesium, curcumin, resveratrol and epigallocatechin-3-gallate were found in experimental animal studies. A reduction in pain related to endometriosis was shown in women using omega-3 and alpha-lipoic acid. Meanwhile, decreasing endometriotic lesion size after the supplementation of omega-3, *N*-acetylcysteine, vitamin C and epigallocatechin-3-gallate was observed in animal and human studies [11]. The rationale for using dietary supplements in the treatment of endometriosis is their potential impact on the pathomechanisms of this disease, as observed in experimental studies. The impact of individual supplements on the potential pathomechanisms of endometriosis is presented in Table 1.

Given that the suffering of women with endometriosis may prompt both them and less experienced doctors to seek alternative treatments, we decided to critically review the studies suggesting the effectiveness of dietary supplements in the treatment of endometriosis.

Thus, the aim of the critical review was to summarize the available data describing the effects of dietary supplements such as vitamin D, vitamin C, vitamin E, zinc, magnesium, selenium, α-LA, omega-3, probiotics, curcumin, resveratrol, propolis, quercetin, NAC, EGCG, DIM and the combination of PEA and PLD used in the treatment of endometriosis.

## 2. Methods

### 2.1. Search Strategy

**Figure 1:**
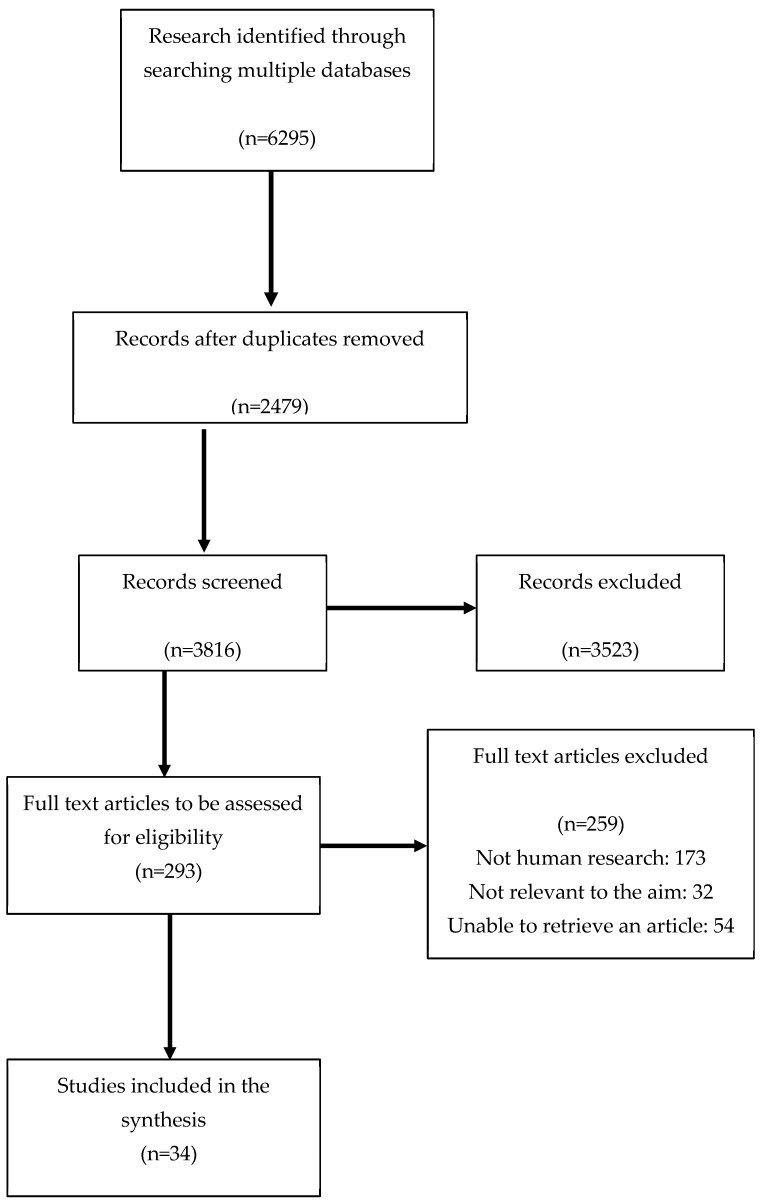
Flow chart of the proceedings in the selection of sources.

The PubMed, Embase, Cochrane, and Web of Science databases were searched for related studies until 15 December 2025. A text search with the following keywords singly or in combination was conducted (in alphabetical order): agnus castus, ALA, alpha-lipoic acid, apple pectins, bilberry powder, bromelain, calcium D-glucorate, calcium L-methylofolate, choline, chromium, chrysin, curcumin, diindolylmethane, endometriosis, endometrioma, epigallocatechin-3-gallate, EGCG, folic acid, iodine, magnesium, milk thistle, *N*-acetyl cysteine, omega-3, palmitoylethanolamide, piper nigrum, polydatin, prebiotics, probiotics, propolis, quercetin, resveratrol, royal jelly, sea buckthorn, selenium, silibinin, silymarin, sulforaphane, synbiotics, Vaccinium myrtillus, vitamin B5, vitamin B6, vitamin B12, vitamin C, vitamin D, vitamin E, zinc, and endometriosis. The final search results were exported into EndNote, and duplicates were removed. The detailed search strategy is shown in Figure 1.

### 2.2. Inclusion and Exclusion Criteria

Accepted studies met the following criteria: (1) analysis of diet supplement use in women with endometriosis, (2) articles published in English, (3) studies involving human participants, and (4) studies including longitudinal studies, and meta-analysis. Papers were excluded if they did not fit into the conceptual framework of the study.

### 2.3. Data Extraction

Data extraction was conducted with the following information: (1) name of the first author, (2) publication year, (3) country, (4) study design, (5) sample size, (6) endometriosis diagnosis, (7) type of dietary supplement used and (8) comparison with a control group. Due to the fact that such a small number of studies were performed among women with endometriosis, and that our manuscript is a narrative review, the quality of the research was not assessed as a meta-analysis.

## 3. Vitamins

### 3.1. Vitamin D

The meta-analysis of nine studies including 976 women with endometriosis and 676 controls showed that the studies assessing the deficiency of vitamin D in women with endometriosis are inconclusive. Three of them showed no differences in vitamin D levels between women with and without endometriosis, while six showed significantly lower levels of this vitamin in women with endometriosis [12]. Vitamin D influences cell differentiation and proliferation by an immunomodulating effect [13]. Its receptors and metabolizing enzymes are found in various immune cells as well as in ovaries and the endometrium [14]. It has also been shown that vitamin D increases anti-inflammatory and decreases pro-inflammatory cytokine release [15]. These effects indicate a potential benefit of vitamin D supplementation in the treatment of endometriosis. We analyzed the results of four available studies that assessed vitamin D supplementation in women with endometriosis. This analysis is presented in Table 2. Only one of the four studies showed the effectiveness of vitamin D supplementation in treating endometriosis-related pain. Furthermore, the administration of a single high dose of vitamin D did not affect the rate of clinical pregnancy in women with endometriosis subjected to an in vitro procedure. Thus, although some studies have shown lower serum vitamin D concentrations in women with endometriosis [16] and an association between low vitamin D consumption in the diet and the higher risk of the development of endometriosis [17], its supplementation does not affect the course of this disease.

### 3.2. Vitamin C

Vitamin C is one of the most important anti-oxidants in the human body [21]. It also has anti-inflammatory and anti-angiogenic effects [22]. Some studies suggested that higher dietary intake of vitamin C decreased the rate of endometriosis diagnosis [23,24]. However, only one randomized placebo-controlled trial, including 245 women with endometriosis aged 28–35 years, assessed the effect of supplementation of 1000 mg vitamin C for 2 months on the outcome of in vitro fertilization. This study showed no significant change in retrieved oocyte, implantation and clinical pregnancy rate between the study and control groups [25].

### 3.3. Vitamin E

The main effects of vitamin E include the inhibition of lipid peroxidation and oxidative stress [26]. Lower serum vitamin E levels in women with moderate-to-severe endometriosis were observed than in women with minimal to mild endometriosis [27]. In the prospective cohort Nurses’ Health Study II performed between 1991 and 2005 a total of 1383 incident cases of laparoscopically confirmed endometriosis were observed among 70,617 women during 735,286-person years of follow-up. Dietary intake of thiamine, folate, vitamin C and vitamin E was inversely associated with the risk of the development of endometriosis, while taking these vitamins in supplement form had no such effect [23]. There is a lack of randomized controlled clinical trials assessing vitamin E supplementation in the treatment of endometriosis.

### 3.4. Vitamins C and E

We analyzed four studies that assessed the supplementation of high doses of both vitamin C and E (Table 3). Three of these studies showed a reduction in the severity of chronic pain, three in pain associated with menstrual cycle, and two in dyspareunia. However, it should be noted that the time of observation in all analyzed studies was short (8 weeks). Therefore, considering the variability of pain intensity in the natural course of endometriosis based on an observation time equal to two menstrual cycles, it is difficult to draw conclusions that this supplementation is effective in the treatment of pain associated with it.

## 4. Microelements

### 4.1. Zinc

Zinc has anti-inflammatory properties. It also plays an important role in reductions in oxidative stress [32,33]. Some studies have shown lower serum zinc levels in women with endometriosis [34,35]. Moreover, lower zinc levels were observed in the ovarian follicular fluid of infertile women with endometriosis than women with tubal infertility [36]. There is a lack of studies assessing zinc supplementation solely in the treatment of endometriosis. So far only one multicenter open-label non-comparative clinical trial, including 346 women with endometriosis aged 27–42 years, assessed zinc supplementation as a part of the oral administration of *N*-acetyl cysteine 600 mg, alpha-lipoic acid 200 mg, bromelain 25 mg and zinc 10 mg daily for 6 months in the treatment of chronic pain associated with endometriosis. This study showed a statistically significant decrease in the pain severity assessed on the basis of the visual analog scale (VAS) after 3- and 6-month therapy periods [37]. No conclusions can be drawn from this study about the benefits of zinc supplementation in the treatment of chronic pain associated with endometriosis.

### 4.2. Magnesium

Magnesium participates in protein and deoxyribonucleic acid (DNA) synthesis, enzyme activity, and neuromuscular excitability [38]. It has been found that magnesium relaxes smooth muscles and may decrease the retrograde menstruation considered to be one of the causes of endometriosis [39]. Moreover, in the results of a prospective cohort study including 70,556 premenopausal US women that assessed consumption of macro and micronutrients based on a 130-item food-frequency questionnaire (FFQ) at baseline and every four years during follow-up, a 14-year period showed an inverse association between magnesium consumption and the occurrence of endometriosis [17]. There is a lack of studies assessing magnesium supplementation solely in the treatment of endometriosis. Currently, only one prospective, randomized, double-blind, placebo-controlled trial assessed the effect of the magnesium sulfate (50 mg in 100 mL saline administered intra venous) (MAG) addition to tincture of opium (TOP) and buprenorphine (BUP) on pain and quality of life in women with dysmenorrhea (106 with diagnosed and 57 with suspected endometriosis). In this study patients were randomized into six subgroups (1:1:1:1:1:1) treated with TOP + MAG, BUP + MAG, TOP + placebo, BUP + placebo, placebo + MAG, and placebo + placebo for the duration of six monthly menstrual periods. There were no significant differences in pain severity and quality of life between subgroups treated with placebo + MAG and placebo + placebo [40].

### 4.3. Selenium

Selenium is an anti-oxidant necessary for intracellular redox reactions by regulation of the enzyme glutathione peroxidase. So far only one triple-blind randomized controlled trial including 64 Iranian women with endometriosis aged 15–49 years assessed the effect of 3 months of 200 μg/day selenium or placebo supplementation on the intensity of dysmenorrhea, dyspareunia, dysuria, dyschezia and noncyclic pain using the VAS and changes in endometrioma size. The study showed a statistically significant decrease in the severity of dysmenorrhea, dyspareunia, dysuria, dyschezia and noncyclic pain and a reduction in endometrioma size in the group using selenium compared to the placebo [41]. However, the small study group size and short time of supplementation do not allow confirmation of this treatment effectiveness.

## 5. Fatty Acids

### 5.1. Alpha-Lipoic Acid (α-LA)

Alpha-lipoic acid (α-LA) has anti-inflammatory and anti-oxidant properties. There is a lack of studies assessing the administration of α-LA alone. We analyzed three studies in which α-LA was one of the components of the used dietary supplements (Table 4). All analyzed studies showed a significant decrease in the severity of chronic pelvic pain, dysmenorrhea and dyspareunia. However, it should be noted that none of these studies were randomized, double-blind, placebo-controlled clinical trials.

### 5.2. Omega-3

Polyunsaturated fatty acids omega-3 have antiproliferative, anti-angiogenic, anti-inflammatory, and anti-apoptotic effects [44]. However, the data from two case–control studies conducted in Northern Italy between 1984 and 1999 including 504 women aged <65 years with a laparoscopically confirmed diagnosis of endometriosis and 504 controls showed no association between fish consumption and endometriosis [45]. We analyzed the results of three available randomized controlled trials (Table 5). Just one of these studies showed the effectiveness of omega-3 fatty acids in treating symptoms caused by endometriosis.

## 6. Probiotics

Probiotics modulating intestinal microbiota improved immunity. Moreover, the *Lactobacillus plantarum*, *Lactobacillus reuteri*, *Bifidobacterium adolescentis* and *Bifidobacterium pseudocatenulatum* contained in probiotics produce vitamin B [48]. These properties may potentially influence the course of endometriosis. We analyzed two available randomized placebo-controlled trials (Table 6). Their results do not allow us to conclude that probiotics are effective in treating pain associated with endometriosis.

## 7. Anti-Oxidants

### 7.1. Curcumin

In vitro and vivo studies showed that polyphenol curcumin has anti-inflammatory, anti-oxidant, anti-mutagenic, anti-metastatic, anti-tumor, and hormonal regulation properties [51]. It has also been found that curcumin inhibits estrogen production and in this way the development of endometriosis [52]. We analyzed two available randomized placebo-controlled trials and one open-label study (Table 7). Their results do not allow us to conclude that curcumin is effective in treating pain associated with endometriosis.

### 7.2. Resveratrol

Resveratrol has anti-inflammatory, anti-oxidant, anti-atherogenic, and anti-angiogenic properties [56]. The experimental studies assessing the effect of resveratrol use in endometriosis showed that resveratrol may inhibit the development of endometriosis [57,58,59,60,61]. One randomized controlled trial and one observational study assessed the addition of resveratrol to contraceptive pills (Table 8).

## 8. Other

### 8.1. Propolis

The flavonoids contained in propolis have anti-inflammatory, anti-oxidant, anti-bacterial, and anti-viral properties [64]. An experimental study showed that chrysin, one of the flavonoids found in propolis, suppresses the phosphatidylinositol 3-kinase (PI3K)/AKT signaling pathway, decreasing the proliferation of endometriotic cells and increasing its apoptosis [65]. Only one study assessed the effect of propolis use in women with endometriosis. This prospective, randomized, placebo-controlled, blind trial including 40 women with laparoscopically diagnosed minimal or mild endometriosis aged 28–36 years treated with 500 mg of bee propolis or placebo daily for 9 months showed that bee propolis was superior to the placebo for pregnancy rate [66]. However, this study is not sufficient to recommend the use of propolis in the treatment of endometriosis.

### 8.2. Quercetin

Quercetin is a flavonoid with a potential effect on apoptosis and anti-estrogenic and progestogenic properties [67,68]. There were no clinical trials assessing the effectiveness of quercetin monotherapy in the treatment of endometriosis. Two studies that assessed quercetin use as a component of fixed combination were described above [46,54], one of them being a randomized placebo-controlled clinical trial confirming the effectiveness of the combined preparation containing quercetin in the treatment of endometriosis. However, it should be noted that quercetin was one of the components of a combined preparation including quercetin, curcumin, parthenium, nicotinamide, 5-methyltetrahydrofolate, and omega-3/6 [46].

### 8.3. N-acetylcysteine (NAC)

Experimental studies found that NAC, the acylated form of cysteine, decreases cell proliferation and its locomotor behavior as well as downregulates inflammatory cytokine activity [69]. There is a lack of randomized placebo-controlled trials assessing the effectiveness of NAC in the treatment of endometriosis. We analyzed data from three available studies including two observational cohort studies and one randomized clinical trial (Table 9). Although two observational studies showed the effectiveness of NAC in treating pain associated with endometriosis, these data are insufficient to recommend its use.

### 8.4. Epigallocatechin-3-gallate (EGCG)

EGCG is bioactive polyphenol especially found in green tea with anti-oxidant and anti-inflammatory properties. Experimental studies showed that EGCG suppresses the estrogen-related activation and proliferation of endometrial cells [73,74,75]. There is a lack of available clinical trials assessing the effectiveness of EGCG in the treatment of endometriosis. We found one registered clinical trial in the database of registered clinical trials: ‘Randomised Double-blinded Placebo Controlled Trial of Green Tea Extract for Endometriosis’. This study was planned to be performed from 2016 to 2022. According to data from 2023, all of the planned 185 women were included in the study with ultrasound-confirmed endometriosis [76]. However, the results of the study have not yet been published. Thus, there is currently no data to recommend EGCG in the treatment of endometriosis.

### 8.5. Diindolylmethane (DIM)

DIM arises with indole-3-carbinol as a result of the digestion of cruciferous vegetables by stomach acid. It has been suggested that DIM may stimulate the production of a less potent, more beneficial form of estrogen known as 2-hydroxyestrone [77]. Only one single-center clinical observational study included an assessment of DIM effectiveness as a supplement to dienogest in endometriosis therapy. This study showed a significantly higher decrease in the severity of chronic pelvic pain associated with endometriosis. However, the cited study included only eight women with endometriosis which is insufficient to determine the effectiveness of DIM [78].

### 8.6. Palmitoilethalonamide/Palmitoylethanolamide (PEA) Combination with Polydatin (PLD)

A structural analog of anandamide *N*-Palmitoylethanolamine (PEA) has anti-inflammatory, immunosuppressive, analgesic, neuroprotective, and anti-oxidant properties [79]. While polydatin (PLD) is a natural glucoside of resveratrol, inhibiting the synthesis and release of pro-inflammatory cytokines and mast cells degranulation as well as modifying eicosanoid synthesis [80]. We analyzed data from three available studies assessing the effects of PEA and PLD supplementation on pain associated with endometriosis including one randomized, double-blind, parallel-group, placebo-controlled clinical trial (Table 10). The cited trial showed that the combination of PEA and PLD is superior to placebo but not to Celecoxib regarding the treatment of pain in endometriosis [81]. The effectiveness of this combination in treating endometriosis-related pain is also suggested by observational studies [82,83]. However, these data are insufficient to recommend the combination of PEA and PLD in the treatment of endometriosis-related pain.

## 9. Should Dietary Supplements Be Recommended for the Treatment of Endometriosis?

The main symptoms of endometriosis are various types of pain, such as chronic pelvic pain, dysmenorrhea, and dyspareunia. Pain significantly impacts the daily functioning, mental state, and social interactions of women with endometriosis. Effective treatment is difficult in clinical practice because the pain experience and response to treatment are individual. The results of numerous experimental studies demonstrating the effectiveness of various dietary supplements in the treatment of endometriosis, as well as the results of studies demonstrating the relationship between vitamin and other dietary intake and the occurrence of endometriosis, may raise hopes not only among women with endometriosis but also among physicians that supplementation will bring beneficial results. These hopes may be supported by research demonstrating the effectiveness of various dietary supplements. This critical review of such studies clearly indicates that there is a lack of evidence supporting the effectiveness of the dietary supplements analyzed in the treatment of endometriosis. In Table 11, we once again summarize the results of randomized placebo-controlled clinical trials assessing the effect of the analyzed dietary supplements on the severity of pain associated with endometriosis. It should be noted that all these studies were single-center and included a small number of participants.

It should be noted that there is a lack of RCT studies assessing the effectiveness of vitamin E, zinc, α-LA, EGCG, and DIM. The supplementation of vitamin C, magnesium, selenium, resveratrol, propolis, NAC and PEA and PLD were assessed in single RCTs. Four RCTs evaluating vitamin D supplementation varied in dose and duration, the number of patients included, and patients’ age and endometriosis severity, as well as endpoints (3: severity of pain; 1: pregnancy rate). Among the four RCTs assessed, supplementation with vitamins C and E was assessed in three studies; the doses, durations, and endpoints were the same across all four, but the number of patients, age, and endometriosis severity varied between studies. Of the two RCTs evaluating omega-3 supplementation, only one assessed its use alone. In two RCTs analyzing probiotics, the effectiveness of supplementation with different strains was assessed. Two RCTs assessed curcumin supplementation, varying in dose and duration; they included different numbers of patients, different ages, and different endometriosis severities, as well as endpoints (1: severity of pain; 1: severity of pain and endometrioma size). Despite being classified as RCTs, the strength of evidence is low due to small group sizes, short observation periods, and the lack of multicenter studies. The characteristics of the analyzed RCTs are presented in Table 12.

In summary, the endpoints in most RCTs were subjective assessments of endometriosis-related pain intensity; some assessed only chronic pain intensity, and some also included dysmenorrhea and dyspareunia. It is also important to note the different doses used for these supplements, which were studied more extensively; the variable duration of intervention; the lack of analysis of endometriosis stages and duration; missing laparoscopic confirmation in some studies; and the lack of analysis of other clinical conditions that may influence pain intensity, such as depression or functional gastrointestinal disorders, could have significantly impacted the results of the analyzed studies.

Despite the hopes raised by the results of experimental and some observational studies as well, their comparison with RCTs, even those conducted in small groups, does not allow us to conclude that any of the supplements analyzed should be included in the standard treatment of women with endometriosis. However, at this stage, the low quality of the RCTs does not allow us to fully deny their effectiveness. Therefore, large, multicenter studies are necessary, especially for supplements that have demonstrated potential efficacy in all types of studies conducted to date. A summary of the effectiveness of the individual supplements analyzed in various types of studies is presented in Table 13.

It should be noted that for a significant part of the dietary supplements discussed in this study, observational studies or RCTs assessing their effectiveness primarily in the treatment of pain caused by endometriosis, in vitro studies have not been conducted; these include zinc, magnesium, selenium, α-LA, probiotics and the combination of PEA and PLD. Some of them, including zinc, selenium, α-LA, and probiotics, have also not been studied in animal models. The rationale for their use was sought based on potential mechanisms of action known from other studies. On the other hand, for supplements such as vitamin D, vitamin C, magnesium, omega-3, curcumin, resveratrol, NAC, and the combination of PEA and PLD, despite encouraging results from in vitro and/or experimental animal studies, RCTs have yielded either negative or inconsistent findings. This may be due to biological diversity, insufficiently low doses, or too-short duration of application, and the use of endpoints that primarily focus on subjective pain assessment.

Considering the limitations of the existing research and the discrepancies between experimental, observational, and RCT studies, well-designed RCTs are essential to confirm or refute the role of dietary supplements in the treatment of endometriosis. These studies should be multicenter and include an appropriate statistically supported study group size, stage of endometriosis, surgical treatment, disease duration, location, standardized doses, potential combinations of supplements with different mechanisms of action, and well-established endpoints (in the case of subjective scales analyzing pain intensity, the use of more than one tool should be considered). Study duration is also important; following the example of many clinical trials, we suggest a minimum of 54 weeks of intervention, with at least 12 weeks of follow-up after treatment, and finally, an assessment of willingness to maintain the intervention after trial completion.

## 10. Limitation of the Review

The main limitation of the review is the lack of multicenter randomized placebo-controlled trials. The second limitation is that all of the studies were performed in small groups. Third is the short duration of all randomized placebo-controlled trials. Fourth, this review did not include publications in languages other than English.

## 11. Conclusions

A detailed analysis of RCTs assessing the effectiveness of dietary supplements on the symptoms of endometriosis showed the lack of RCTs for vitamin E, zinc, α-LA, EGCG, and DIM. Moreover, single RCTs for vitamin C, magnesium, resveratrol, NAC, PEA, and PLD, despite experimental studies, confirmed their effectiveness. Furthermore, single RCTs on selenium, propolis, and quercetin confirmed their effectiveness in treating pain related to endometriosis. Finally, RCTs for vitamin D, omega-3, curcumin, and probiotics have yielded mixed results; only RCTs assessing the effectiveness of vitamin C and E have confirmed it.

Despite encouraging observations from experimental studies, the results of RCTs are less encouraging and do not allow for the formulation of recommendations concerning the use of supplements in the treatment of endometriosis symptoms according to EBM.

## Figures and Tables

**Table 1:** The impact of dietary supplements on the pathomechanisms of endometriosis.

Pathomechanism	Dietary Supplements
Inflammation	Vitamin D, vitamin C, zinc, alpha-lipoic acid, omega-3, curcumin, resveratrol, propolis, *N-*acetylcysteine, epigallocatechin-3-gallate, palmitoilethalonamide/palmitoylethanolamide combination with polydatin
Oxidative stress	Vitamin C, vitamin E, zinc, selenium, alpha-lipoic acid, curcumin, resveratrol, propolis, epigallocatechin-3-gallate, palmitoilethalonamide/palmitoylethanolamide combination with polydatin
Immune dysregulation	Vitamin D, probiotics, curcumin, palmitoilethalonamide/palmitoylethanolamide combination with polydatin
Estrogen signaling	Curcumin, epigallocatechin-3-gallate, diindolylmethane
Angiogenesis	Vitamin C, omega-3, resveratrol
Apoptosis	Omega-3, quercetin, propolis
Cell proliferation	*N-*acetylcysteine, omega-3, propolis

**Table 2:** The results of studies that assessed the effect of vitamin D supplementation in women with endometriosis.

Author	Methods	Characteristics of Population	Type of Study	Results
Almassinokiani et al. [14], 2016	Oral administration of 50,000 IU vitamin D or placebo weekly for 12 weeks. Assessment of pain severity based on VAS test at 24 weeks after surgical treatment.	39 women aged 15–40 years with endometriosis diagnosed and treated by laparoscopy, with scores of at least 3 for dysmenorrhea and/or pelvic pain at 8 weeks after surgical treatment	double-blind randomized clinical trial 1:1	No significant effect on severity of dysmenorrhea and/or pelvic pain.
Nodler et al. [18], 2020	Oral administration of 2000 IU vitamin D or 1000 mg fish oil or placebo daily for 6 months. Pain assessment on the basis of VAS.	69 women aged 12–25 years with surgically confirmed endometriosis and pelvic pain	double-blind, randomized, placebo-controlled trial 1:1:1	In both of the intervention arms the decrease in pain severity was statistically insignificant compared to the placebo.
Mehdizadehkashi et al. [19], 2021	Oral administration of 50,000 IU vitamin D or placebo every 2 weeks for 12 weeks. Pain assessment on the basis of VAS.	50 women aged 18–40 years with endometriosis	randomized, double-blind, placebo-controlled trial 1:1	The significant decrease in pelvic pain severity was found in the vitamin D group compared to the placebo.
Somigliana et al. [20], 2021	Single oral administration of 600,000 IU vitamin D or placebo. Assessment of changes in ratio of clinical pregnancy.	83 normal weight women aged 18–39 years with endometriosis with preserved ovarian reserve and low vitamin D level undergoing in vitro fertilization	2-center randomized superiority double-blind placebo-controlled trial	There were no differences in the improvement of the rate of clinical pregnancy between vitamin D and placebo group.

**Table 3:** The results of studies that assessed the effect of vitamin C and E supplementation in women with endometriosis.

Author	Methods	Characteristics of Population	Type of Study	Results
Santanam et al. [28], 2013	Oral administration of 1200 IU vitamin E and 1000 mg vitamin C combination (group A) or placebo (group B) daily for 8 weeks. Changes in severity of chronic pelvic pain, dysmenorrhea, dyspareunia using an arbitrary scale of “none, mild, moderate and severe” levels of pain.	59 women aged 19–41 years with pelvic pain and history of endometriosis or infertility	randomized placebo-controlled trial 46:13	A significantly higher decrease in severity of chronic pain was found in group A than group B, while the changes in dysmenorrhea and dyspareunia did not differ between study groups.
Ibrahim et al. [29], 2022	Oral administration of 1200 IU vitamin E and 1000 mg vitamin C combination (group A) or placebo (group B) daily for 8 weeks. Changes in severity of chronic pelvic pain, dysmenorrhea, dyspareunia using VAS.	100 women aged 19–41 years with endometriosis	prospective randomized placebo-controlled trial 1:1	A significantly higher decrease in severity of dysmenorrhea was found in group A than group B, while the changes in chronic pelvic pain and dyspareunia did not differ between study groups.
Alnaggar et al. [30], 2022	Oral administration of 1200 IU vitamin E and 1000 mg vitamin C combination (group A) or placebo (group B) daily for 8 weeks. Changes in severity of chronic pelvic pain, dysmenorrhea, dyspareunia using VAS.	60 women aged 19–41 years diagnosed with endometriosis	randomized placebo-controlled trial 1:1	A significantly higher decrease in severity of chronic pelvic pain, dysmenorrhea and dyspareunia was found in group A than group B.
Amini et al. [31], 2021	Oral administration of 800 IU vitamin E and 1000 mg vitamin C combination (group A) or placebo (group B) daily for 8 weeks. Changes in severity of chronic pelvic pain, dysmenorrhea, dyspareunia using VAS.	60 women aged 15–45 years diagnosed with endometriosis	randomized triple-blind placebo-controlled clinical trial 1:1	A significantly higher decrease in severity of chronic pelvic pain, dysmenorrhea and dyspareunia was found in group A than group B.

**Table 4:** The results of studies assessing the effect of α-LA as one of the supplementation components in women with endometriosis.

Author	Methods	Characteristics of Population	Type of Study	Results
Lete et al. [37], 2018	*N*-acetyl cysteine 600 mg, alpha-lipoic acid 200 mg, bromelain 25 mg and zinc 10 mg combination daily for 6 months. Changes in the severity of chronic pelvic pain using VAS score.	346 women aged 27–42 years with endometriosis	multicenter, open-label, non-comparative clinical trial	The severity of chronic pelvic pain decreased significantly after 3 months and 6 months.
De Leo et al. [42], 2019	Oral administration of nutraceutical product (Pelvinox^®^) containing alpha-lipoic acid 800 mg, palmitoilethalonamide 600 mg and myrrh 200 mg) daily for 6 months. Changes in mean diameter of the endometrioma in ultrasonography, severity of chronic pelvic pain, dyspareunia and dysmenorrhea.	60 women aged 20–39 years with endometrioma and chronic pelvic pain	multicenter, open-label, non-comparative clinical trial	A significant decrease in severity of dysmenorrhea, dyspareunia and chronic pelvic pain after the 6-month period was found. However, there was no significant change in the mean diameter of the endometrioma.
Caruso et al. [43], 2015	Oral administration of alpha-lipoic acid 600 mg and palmitoilethalonamide 600 mg combination (in total dose) daily for 9 months. Changes in the severity of chronic pelvic pain level, dyspareunia and dysmenorrhea using VAS, quality of life (QoL), the short form-36, female sexual function index and female sexual distress scale.	56 women aged 18–31 years (at baseline); 51 (3rd month); 46 (6th month); 41 (9th month) with endometriosis and a VAS test score of more than 5	one center open-label, non- comparative clinical trial	A statistically significant decrease in analgesics usage, severity of chronic pain, dysmenorrhea and dyspareunia as well as the improvement of female sexual function index, female sexual distress scale and quality of life were found after 9 months of follow-up.

**Table 5:** The results of studies assessing the effect of omega-3 supplementation in women with endometriosis.

Author	Methods	Characteristics of Population	Type of Study	Results
Nodler et al. [18], 2020	Oral administration of 2000 IU vitamin D or 1000 mg fish oil or placebo daily for 6 months. Pain assessment on the basis of VAS.	69 women aged 12–25 years with surgically confirmed endometriosis and pelvic pain	double-blind, randomized, placebo-controlled trial 1:1:1	In both of the intervention arms the decrease in pain severity was statistically insignificant compared to the placebo.
Signorile et al. [46], 2018	Oral administration of dietary supplement combination including 1002 mg linoleic acid (omega-3), 432 mg alpha linolenic acid (omega-3), 172.8 mg linoleic acid (omega 6), 200 mg quercetin, 20 mg nicotinamide, 400 mcg 5-methyltetrahydrofolate calcium salt, 20 mg titrated turmeric, 19.5 mg titrated parthenium (group A), dietary supplement combination including 1002 mg linoleic acid (omega-3), 432 mg alpha linolenic acid (omega-3), 172.8 mg linoleic acid (omega 6) and 400 mcg 5-methyltetrahydrofolate calcium salt (group B) or placebo (group C) twice daily for 3 months. The intensity of headache, cystitis, muscular pain or fibromyalgia, irritable colon, dysmenorrhea, dyspareunia, chronic pelvic pain, were measured using the visual analog scale (VAS).	90 women aged mean 35 years with IV stage endometriosis	randomized placebo-controlled trial 1:1:1	A significant reduction in the symptoms of endometriosis patients treated with the dietary composition compared to the controls.
Abokhrais et al. [47], 2020	Oral administration of two capsules containing 1000 mg (2000 mg total daily dose) of O-PUFA (Omega-3-acid ethyl ester PUFA) (A) or placebo (olive oil) (B) daily for 8 weeks. Changes in pain were assessed based on Brief Pain Inventory.	27 women aged 18–50 years with endometriosis and pain greater than or equal to four in NRS for two or more weeks prior to randomization	two-arm parallel double-blind randomized controlled trial 1:1	There were no statistical changes in the pain severity during observation in both groups A and B.

**Table 6:** The results of studies assessing the effect of probiotic supplementation in women with endometriosis.

Author	Methods	Characteristics of Population	Type of Study	Results
Itoh et al. [49], 2011	Oral administration of tablets containing 100 mg of *L. gasseri* OLL2809 in total (group A) or placebo (group B) daily for 12 weeks. Changes in dysmenorrhea and chronic pain severity using VAS and VRS.	62 women aged 18–45 years with endometriosis	randomized, double-blind, placebo-controlled study 1:1	Changes in VAS in pain intensity at menstrual period were significantly higher in group A than B after 2 and 3 months of therapy. Changes in VRS in dysmenorrhea were higher in group A than B after 3 months of therapy. Change in VAS in pain intensity at non-menstrual period did not differ between groups A and B.
Khodaverdi et al. [50], 2019	Oral administration of LactoFem ^®^ tablets containing 109 colonies of four different lactobacillus strains (*L. acidophilus, L. plantarum, L. fermentum and L. gasseri*) (A) or placebo (B) daily for 8 weeks. Changes in intensity of pain using VAS for dysmenorrhea, dyspareunia and chronic pelvic pain.	32 women aged 18–45 years with surgically and pathologically diagnosed endometriosis	randomized pilot placebo-controlled trial 1:1	There were no significant differences between baseline and week 12 in intensity of dyspareunia, dysmenorrhea, chronic pelvic pain and overall pain score between groups A and B.

**Table 7:** The results of studies assessing the effect of curcumin supplementation in women with endometriosis.

Author	Methods	Characteristics of Population	Type of Study	Results
Gudarzi et al. [53], 2024	Oral administration of curcumin (500 mg) (A) or placebo (B) twice daily for 8 weeks. Changes in painful symptoms of endometriosis using VAS.	68 women aged 27–40 years with endometriosis	triple-blind randomized controlled trial 1:1	There were no statistically significant differences in changes in usual pain, pain at its worst and visual pain between groups A and B.
Fadin et al. [54], 2020	Oral administration of combination consisting of 200 mg of quercetin + 210 mg of turmeric (of which 200 mg of curcuminoids) + 150 mg of *N*-acetylcysteine (1 tablet of ALLIENDo^®^ a day) for 2 months. Changes in the use of NSAIDs, and severity of dysmenorrhea, pelvic pain and dyspareunia using VAS.	33 women aged 18–50 years with endometriosis	open-label, single-center, versus historical control study	Significant reduction in NSAIDs use and severity of dysmenorrhea, pelvic pain and dyspareunia after 2 months therapy period.
Sargazi-Taghazi et al. [55], 2025	Oral administration of 80 mg/day nanocurcumin soft gel capsules (80 mg/day) or placebo with 2 mg/day dienogest for 8 weeks. Changes in painful symptoms of endometriosis using VAS and endometrioma size.	86 women aged 18–45 years with stage 2–3 pelvic endometriosis and moderate to severe pain	randomized, double-blind, placebo-controlled trial 1:1	Significantly greater decrease in severity of dysmenorrhea, dyspareunia, chronic pelvic pain and dyschezia compared to placebo. There were no significant differences in endometrioma size between study groups.

**Table 8:** The results of studies assessing the effect of resveratrol supplementation added to the treatment with contraceptive pills in women with endometriosis.

Author	Methods	Characteristics of Population	Type of Study	Results
Mendes da Silva et al. [62], 2017	Oral administration of 40 mg/d resveratrol with monophasic contraceptive pill or placebo with monophasic contraceptive pill for 42 days. Changes in painful symptoms of endometriosis using VAS.	44 women aged 20–50 years with laparoscopically diagnosed endometriosis	double-blind randomized controlled trial 1:1	There was no statistically significant difference in change in pain between resveratrol and placebo groups.
Maia et al. [63], 2012	Oral administration of 30 mg/d resveratrol with a contraceptive pill containing drospirenone and ethinylestradiol for 6 months. Changes in dysmenorrhea and pelvic pain scores.	12 women aged 22–37 years with endometriosis failed to obtain pain relief during use of an oral contraceptive containing drospirenone + ethinylestradiol	open-label, single-center study	Significant decrease in pain scores after 6-month therapy period.

**Table 9:** The results of studies assessing the effect of NAC supplementation in women with endometriosis.

Author	Methods	Characteristics of Population	Type of Study	Results
Anastasi et al. [70], 2023	Oral administration of 3 tablets composed of 600 mg *N*-acetyl cysteine (NAC) daily (1800 mg total daily dose) for 3 consecutive days of the week for 3 months. Changes in pregnancy occurrence, dysmenorrhea, dyspareunia, noncyclic chronic pelvic pain using VAS and size of ovarian endometriomas using transvaginal ultrasound scan.	125 women aged 18–45 years with a clinical/histological diagnosis of endometriosis	prospective observational single-cohort study	Significant improvement in the severity of dysmenorrhea, dyspareunia and chronic pelvic pain as well as decrease in size of the endometriomas. Moreover, the use of NSAIDs decreased significantly. In addition, 39 of 52 patients with reproductive desire were pregnant within 6 months of starting therapy.
Porpora et al. [71], 2013	Oral administration of 3 tablets composed of 600 mg *N*-acetyl cysteine (NAC) daily (1800 mg total daily dose) for 3 consecutive days of the week (A) or no treatment (B) for 3 months. Changes in pregnancy rate and severity of dysmenorrhea, dyspareunia, noncyclic chronic pelvic pain using VAS, cyst size and quantity using ultrasonography.	92 women aged 25–40 years with an ultrasound-confirmed diagnosis of endometrioma	observational cohort study 1:1	Significantly higher decrease in severity of dysmenorrhea, dyspareunia, noncyclic chronic pelvic pain was observed in group A than group B. In addition, mean decrease in diameter and volume of endometriomas were higher in group A than group B. There was no significant difference in pregnancy rate between groups A and B.
Asgari et al. [72], 2022	Oral administration of 3 tablets composed of 600 mg *N*-acetyl cysteine (NAC) daily (1800 mg total daily dose) for 3 consecutive days of the week for 3 months in addition to oral contraceptive pills containing 30 mcg Ethinyl estradiol + 0.15 mg levonorgestrel (OCP) daily or OCP as monotherapy for 6 months. Changes in chronic pelvic pain using VAS and endometrioma recurrence.	100 women aged 24–35 years with stage IV endometriosis	randomized placebo-controlled trial 1:1	There were no differences between study groups in the decrease in severity of chronic pelvic pain and recurrence of endometrioma.

**Table 10:** The results of studies assessing the effect of PAE and PLD supplementation in women with endometriosis.

Author	Methods	Characteristics of Population	Type of Study	Results
Cobellis et al. [81], 2011	Oral administration combination of 400 mg PEA and 40 mg transpolydatin twice daily for 3 months (A group), placebo for 3 months (B group) or a single course of Celecoxib (200 mg twice a day for 7 consecutive days) (C group). Changes in severity of dysmenorrhea, chronic pelvic pain, dyspareunia using VAS.	61 women aged 24–41 years with laparoscopic diagnosed endometriosis	randomized, double-blind, parallel-group, placebo-controlled clinical trial 1:1:1	In group A a significant higher decrease in severity of dysmenorrhoea, dyspareunia and pelvic pain than group B was observed. However, these changes were significantly higher in group C than in groups A and B.
Giugliano et al. [82], 2013	Oral administration of 400 mg PEA and 40 mg transpolydatin twice daily for 30 (A1 and B1 subgroups), 60 (A2 and B2 subgroups) and 90 (A3 and B3 subgroups) days. Changes in severity of dysmenorrhea, chronic pelvic pain, dyspareunia and dyschezia using VAS.	47 women aged 24–45 years with endometriosis-related pain according to the endometriosis localization: recto-vaginal septum (group A *n* = 19) or ovary (group B, *n* = 28)	prospective observational study	In both study groups significantly decreased severity of chronic pelvic pain, dysmenorrhea, dyspareunia and dyschezia were found during observation.
Stochino Loi et al. [83], 2019	Oral administration of 600 mg PEA twice daily for 10 days followed by 400 mg PEA and 40 mg PLD twice daily for 80 days. Changes in severity of chronic pelvic pain, dyspareunia, dysmenorrhea, dyschezia, dysuria using VAS. The data was obtained after 10, 30, 60, 90 days and 30 days’ follow-up.	30 women aged 18–48 years with laparoscopic diagnosis of endometriosis	open-label pilot study	Significantly decreased severity of chronic pelvic pain, dysmenorrhea, dyspareunia, and dyschezia after 90 days of treatment was found. No significant change in severity of dysuria was shown. While after 30 days’ follow-up a slight increase in VAS mean scores of all evaluated variables was observed.

**Table 11:** A summary of randomized placebo-controlled clinical trials on supplement use in the treatment symptoms of endometriosis and its results.

Supplement	Total Number of RCT Studies	Number of RCT Studies Confirmed Effectiveness	Number of RCT Studies No Confirmed Effectiveness
vitamin D	4 [14,18,19,20]	1 [18]	3 [14,19,20]
vitamin C	1 [25]	0	1 [25]
vitamin E	0	0	0
vitamins C and E	4 [26,27,28,29,30,31]	4 [26,27,28,29,30,31]	0
zinc	0	0	0
magnesium	1 [40]	0	1 [40]
selenium	1 [41]	1 [41]	0
α-LA	0	0	0
omega-3	3 [18,46,47]	2 [18,46]	1 [47]
probiotics	2 [49,50]	1 [49]	1 [50]
curcumin	2 [53,55]	1 [53]	1 [55]
resveratrol	1 [62]	0	1 [62]
propolis	1 [66]	1 [66]	0
quercetin	1 [46]	1 [46]	0
NAC	1 [72]	0	1 [72]
EGCG	0	0	0
DIM	0	0	0
PEA and PLD	1 [81]	0	1 [81]

**Table 12:** The characteristics of the analyzed RCTs.

Supplement	Dosage	Duration	Patient Population	Endpoints and Effectiveness (Yes or No)
vitamin D	50,000 IU or placebo weekly	12 weeks	39 women aged 15–40 years with laparoscopy diagnosed and treated endometriosis	Severity of dysmenorrhea and pelvic pain based on VAS score [14]. No
	2000 IU or 1000 mg fish oil or placebo daily	6 months	69 women aged 12–25 years with surgically confirmed endometriosis	Severity of pain based on VAS score [18] No
	50,000 IU or placebo every 2 weeks	12 weeks	50 women aged 18–40 years with endometriosis	Severity of pain based on VAS score [19] Yes
	600,000 IU or placebo single dose		83 normal weight women aged 18–39 years with endometriosis with preserved ovarian reserve and low vitamin D level undergoing in vitro fertilization	Rate of clinical pregnancy [20] No
vitamin C	1000 mg or placebo daily	2 months	245 women with endometriosis aged 28–35 years	Retrieved oocyte, implantation and clinical pregnancy rate [25] No
vitamins C and E	1000 mg vitamin C and 1200 IU vitamin E or placebo daily	8 weeks	59 women aged 19–41 years with pelvic pain and history of endometriosis or infertility	Severity of chronic pain, dysmenorrhea and dyspareunia based on VAS score [28] Partially yes
	1000 mg vitamin C and 1200 IU vitamin E or placebo daily	8 weeks	100 women aged 19–41 years with endometriosis	Severity of chronic pain, dysmenorrhea and dyspareunia based on VAS score [29] Partially yes
	1000 mg vitamin C and 1200 IU vitamin E or placebo daily	8 weeks	60 women aged 19–41 years diagnosed with endometriosis	Severity of chronic pain, dysmenorrhea and dyspareunia based on VAS score [30] Yes
	1000 mg vitamin C and 800 IU vitamin E or placebo daily	8 weeks	60 women aged 15–45 years diagnosed with endometriosis	Severity of chronic pain, dysmenorrhea and dyspareunia based on VAS score [31] Yes
magnesium	Magnesium sulfate (50 mg in 100 mL saline administered intravenously) (MAG) addition to tincture of opium (TOP) and buprenorphine (BUP) or placebo + MAG or placebo + placebo	Six monthly menstrual periods	106 women with diagnosed and 57 with suspected endometriosis	Severity of pain and quality of life [40] No
selenium	200 μg/day selenium or placebo	3 months	64 women with endometriosis aged 15–49 years	Intensity of dysmenorrhea, dyspareunia, dysuria, dyschezia and noncyclic pain based on VAS score and changes in endometrioma size [41] Yes
omega-3	2000 IU vitamin D or 1000 mg fish oil or placebo daily	6 months	69 women aged 12–25 years with surgically confirmed endometriosis	Severity of pelvic pain based on VAS [18] No
	Dietary supplement combination including 1002 mg linoleic acid (omega-3), 432 mg alpha linolenic acid (omega-3), 172.8 mg linoleic acid (omega 6), 200 mg quercetin, 20 mg nicotinamide, 400 mcg 5-methyltetrahydrofolate calcium salt, 20 mg titrated turmeric, 19.5 mg titrated parthenium or dietary supplement combination including 1002 mg linoleic acid (omega-3), 432 mg alpha linolenic acid (omega-3), 172.8 mg linoleic acid (omega 6) and 400 mcg 5-methyltetrahydrofolate calcium salt] or placebo twice daily		90 women aged 35 years on avarage with IV stage endometriosis	The intensity of headache, cystitis, muscular pain or fibromyalgia, irritable colon, dysmenorrhea, dyspareunia, and chronic pelvic pain based on VAS [46] Yes
	Two capsules containing 1000 mg (2000 mg total daily dose) of O-PUFA (Omega-3-acid ethyl ester PUFA) or placebo (olive oil) daily	8 weeks	27 women aged 18–50 years with endometriosis	Severity of pain based on the Brief Pain Inventory [47] No
probiotics	Tablets containing 100 mg of *L. gasseri* OLL2809 in total or placebo daily	12 weeks	62 women aged 18–45 years with endometriosis	Severity of dysmenorrhea and chronic pain based on VAS and VRS score [49] Partially yes
	LactoFem^®^ tablets containing 109 colonies of four different lactobacillus strains (*L. acidophilus*, *L. plantarum*, *L. fermentum* and *L. gasseri)* or placebo daily	8 weeks	32 women aged 18–45 years with surgically and pathologically diagnosed endometriosis	Severity of dysmenorrhea, dyspareunia and chronic pelvic pain based on VAS score [50] No
curcumin	500 mg curcumin or placebo twice daily	8 weeks	68 women aged 27–40 years with endometriosis	Painful symptoms of endometriosis based on VAS score [53] No
	80 mg/day nanocurcumin soft gel capsules (80 mg/day) or placebo with 2 mg/day dienogest	8 weeks	86 women aged 18–45 years with stage 2–3 pelvic endometriosis	Painful symptoms of endometriosis based on VAS score and endometrioma size [55] Yes—pain severity/ No—endometrioma size
resveratrol	40 mg/d resveratrol with monophasic contraceptive pill or placebo with monophasic contraceptive pill	42 days	44 women aged 20–50 years with laparoscopically diagnosed endometriosis	Painful symptoms of endometriosis based on VAS score [62] No
propolis	500 mg of bee propolis or placebo daily	9 months	40 women with laparoscopically diagnosed minimal or mild endometriosis aged 28–36 years	Pregnancy rate [66] Yes
quercetin	Dietary supplement combination including 1002 mg linoleic acid (omega-3), 432 mg alpha linolenic acid (omega-3), 172.8 mg linoleic acid (omega 6), 200 mg quercetin, 20 mg nicotinamide, 400 mcg 5-methyltetrahydrofolate calcium salt, 20 mg titrated turmeric, 19.5 mg titrated parthenium or dietary supplement combination including 1002 mg linoleic acid (omega-3), 432 mg alpha linolenic acid (omega-3), 172.8 mg linoleic acid (omega 6) and 400 mcg 5-methyltetrahydrofolate calcium salt] or placebo twice daily	3 months	90 women aged 35 years on avarage with IV stage endometriosis	The intensity of headache, cystitis, muscular pain or fibromyalgia, irritable colon, dysmenorrhea, dyspareunia, and chronic pelvic pain based on VAS [46] Yes
NAC	3 tablets composed of 600 mg *N*-acetyl cysteine (NAC) daily (1800 mg total daily dose) for 3 consecutive days of the week in addition to oral contraceptive pills containing 30 mcg Ethinyl estradiol + 0.15 mg levonorgestrel (OCP) daily or OCP as monotherapy	3 months	100 women aged 24–35 years with stage IV endometriosis	Severity of chronic pelvic pain based on VAS score and endometrioma recurrence [72] No
PEA and PLD	Combination of 400 mg PEA and 40 mg transpolydatin twice daily for 3 months or placebo or a single course of Celecoxib (200 mg twice a day for 7 consecutive days)	3 months	61 women aged 24–41 years with laparoscopically diagnosed endometriosis	Severity of dysmenorrhea, chronic pelvic pain, and dyspareunia based on VAS score [81] No

**Table 13:** Effectiveness of supplements in vitro, animal, observational, and RCT studies.

Supplement	In Vitro	Animal	Observational	RCT
vitamin D	Decreases invasion and proliferation of endometriotic lesions without affecting apoptosis [84,85,86,87]	Regression of endometriotic implants [88,89,90,91]	Missing	4 [14,18,19,20] 3 no confirmed effectiveness [14,19,20]
vitamin C	Suppresses the growth of endometriotic lesions [92]	The prevention and regression of endometriotic implants [93] reduce the volumes and weights of the endometriotic cysts in dose-dependent manner [22]	Missing	1 no confirmed effectiveness
vitamin E	Inhibited proliferation [94]	Lack of effectiveness in the mouse model of endometriosis [74]	Missing	Missing
vitamins C and E	Missing	Missing	Missing	4 showed an effectiveness [26,27,28,29,30,31]
zinc	Missing	Missing	Missing	Missing
magnesium	Missing	Changes in the vascular endothelial growth factor (VEGF) in uterine tissue [95]	Missing	One that has not proven effectiveness [40]
selenium	Missing	Missing	Missing	One that showed effectiveness [41]
α-LA	Missing	Missing	3 studies showed effectiveness in combined therapy [37,42,43]	Missing
omega-3	Suppressive effect on the in vitro survival of endometrial cells reducing the inflammatory response and modulating cytokine function [96]	Significant decrease in size of the implants of endometriosis in rats [97]	Missing	2 out of 3 studies showed effectiveness [18,46,47]
probiotics	Missing	Missing	Missing	1 out of 2 studies showed effectiveness [49,50]
curcumin	The number of endometriotic stromal cells was reduced, and cell growth slowed [52]	Inhibited MMP-2 activity and endometriosis progression [98]	One that showed effectiveness [54]	1 out of 2 studies showed effectiveness [53,55]
resveratrol	Reduced proliferation, migration, and invasiveness of human-cultured EcESC [99]	Reduction in endometriotic implant size [60,100,101,102]	One that showed effectiveness [63]	One that has not proven effectiveness [62]
propolis	Decrease the proliferation of endometriotic cells and increase their apoptosis [65]	Missing	Missing	One that showed effectiveness [66]
quercetin	Inhibition the proliferation and induced the cell cycle arrest in VK2/E6E7 and End1/E6E7 cells. Furthermore, it induced cell apoptosis with DNA fragmentation, loss of mitochondrial membrane potential and reactive oxygen species production [67]	Antiproliferative and anti-inflammatory effects on the endometriosis autoimplanted mouse model [67]	One with a combination of NAC showed effectiveness [54]	One that showed effectiveness in combined therapy [46]
NAC	Inhibition of proliferation [22]	Reduction in endometrioma mass, by switching cell behavior from proliferation toward differentiation, and decreases both tissue inflammation and cell invasiveness [69]	One that showed effectiveness [70,71]	One that has not proven effectiveness [72]
EGCG	Inhibition of the angiogenesis and suppression of VEGFC/VEGFR2 expression and signaling pathway in experimental endometriosis [103]	Inhibition of the development of experimental endometriosis through anti-angiogenic effects [94]	Missing	Missing
DIM	Missing	Decrease in the volume of the lesion in the mouse model [104]	One in combination with dienogest showed effectiveness [78]	Missing
PEA and PLD	Missing	Decrease in size of the implants and the severity of histological marks of endometriotic cysts in rat models [105]	Two showed effectiveness [82,83]	One that has not proven effectiveness [81]

## Data Availability

No new data were created or analyzed in this study. Data sharing is not applicable to this article.

## Abbreviations

The following abbreviations are used in this manuscript:

ACOG	American College of Obstetricians and Gynecologists
AKT	Protein kinase B
ASRM	American Society for Reproductive Medicine
α-LA	Alpha-lipoic acid
BUP	Buprenorphine
DIM	Diindolylmethane
EGCG	Epigallocatechin-3-gallate
FFQ	Food-frequency questionnaire
GNRH	Gonadoliberin
MAG	Magnesium
NAC	*N*-acetylcysteine
NICE	National Institute for Health and Care
NRS	Numerical rating scale
OCP	Oral contraceptive pill
PEA	*N*-Palmitoylethanolamine
PI3K	Phosphatidylinositol 3-kinase
PLD	Polydatin
PUFA	Polyunsaturated fatty acids
TOP	Tincture of opium
VAS	Visual analog scale
VRS	Verbal rating scale
